# Supplementary material for: Single-molecule imaging and quantification of the immune-variant adhesin VAR2CSA on knobs of Plasmodium falciparum-infected erythrocytes
Source: Commun Biol. 2019 May 8;2:172. doi: 10.1038/s42003-019-0429-z (PMC6506540; doi:10.1038/s42003-019-0429-z)
Supplement: Supplementary file 1 — Supplementary Information [file 42003_2019_429_MOESM1_ESM.pdf]

Supplementary Table 1. Primers used in this study. Red, sequences used for InFusion cloning. Underlined, restriction sites.

Primer name	sequence
var2csa 5'UTR for -360	C CAA TGG CCC CTT TCC CCA TTT TGT ATA ATA T TAT GTT CCG
var2csa DBL1x rev +181	GAG TGC AGT AAG TTG GCG CGC ATC TGA ATG ATT CAC GGT AA TTT TAC
var2csa DBL1x for +181	GCT GCA GCC GAA GAT ACT AGT TCT GGA AAG TAT GAT CCT TGT G
var2csa DBL1x rev +756	TTT TTT TAC AAA ATG CAT TTG CGG CAC AAT TCG AAG
mEos-2x-DBL1x-for	GTG AAT CAT TCA GAT TCA GCC ATT AAG CCT GAT ATG
mEos-2x-DBL1x-rev	ATC ATA CTT TCC AGA TCG TCT GGC ATT GTC AGG C
P1-5'UTR integration for -332	AAA ATA TTT GGT GTC TTT ATA AGC
P2-3' integration rev +833	CTA TCC ATT CTG TTA ACC ACC
P3	TT TGT GGAC CAC TGC ATT GAG
P4	GT ACC ATC ACC ATC AAT AAC G
P5	CTA TAG AAG TGG AGG TGA TGG
P6	AC TTC CAT CAG ATG AAT TTT GCT GAC
P7	ACC ATT CCT TTT GGT ATT GCG
P8	CC TTT ATG GAT ATC AAG TAC ACG
var2csa-60 guide for	TAA GTA TAT AAT ATT TCC AGA ATC TGA ATG ATT CA GTT TTA GAG CTA GAA
var2csa-60guide rev	TTC TAG CTC TAA AAC TG AAT CAT TCA GAT TCT GGA AAT ATT ATA TAC TTA
var2csa-2588guide for	TAA GTA TAT AAT ATT T GAA AAA CGT AAA AAA TGG TGTT TTA GAG CTA GAA
var2csa-2588guide rev	TTC TAG CTC TAA AAC A CCA TTT TTT ACG TTT TTC A AAT ATT ATA TAC TTA
var2csa-2310 guide for	TAA GTA TAT AAT ATT A AAA TTT TGG GAG ATG GAG T GTT TTA GAG CTA GAA
var2csa-2310 guide rev	TTC TAG CTC TAA AAC A CTC CAT CTC CCA AAA TTT T AAT ATT ATA TAC TTA

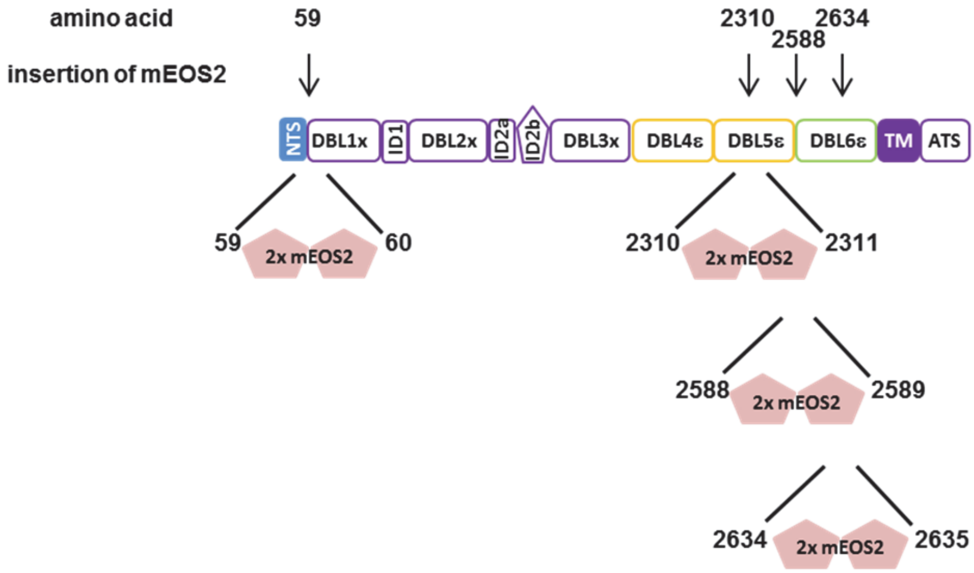

Supplementary Fig. 1 Schematic illustration showing the domain structure of VAR2CSA and the different sites where two copies of mEOS2 were inserted.

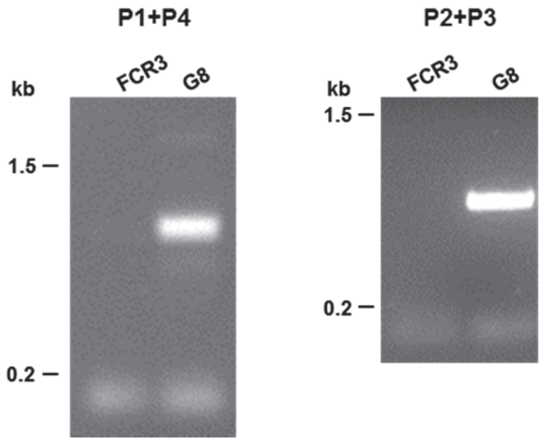

Supplementary Fig. 2 Uncropped gel images of gels presented in the left panel of Fig. 1b. Genomic DNA from the *P. falciparum* strain FCR3 and the genetically engineered parasite line G6 were analyzed by PCR using the primer pairs indicated above the gels. The primer pairs are explained in the legend of Figure 1 and the supplementary Table 1. A size marker is indicated in kilo base pairs (kb).

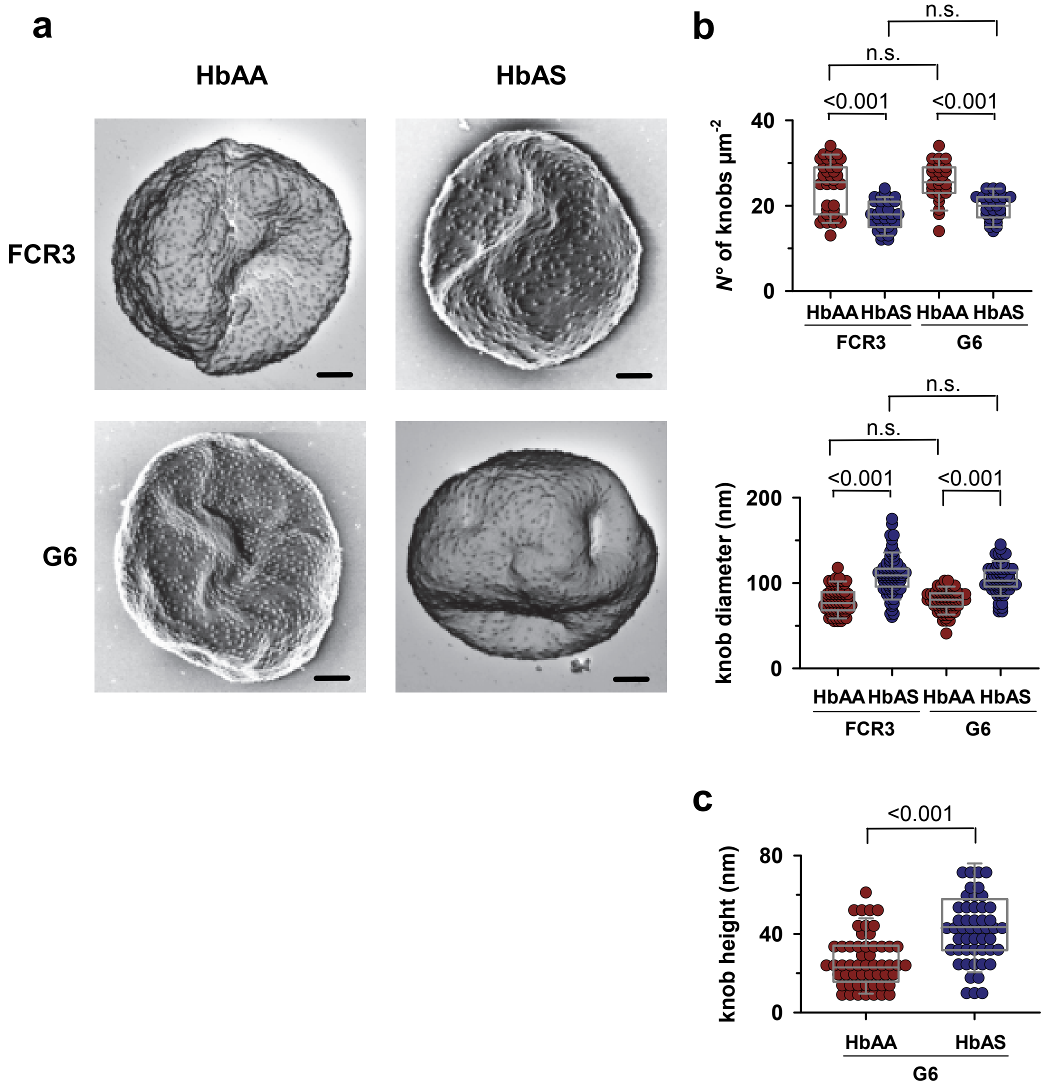

Supplementary Fig. 3 Knob morphology in parasitized HbAA and HbAS erythrocytes. **a** Representative scanning electron microscopic images of HbAA and HbAS erythrocytes infected with the parental $FCR3^{CSA}$ strain and the G6 mutant encoding $VAR2CSA^{mEOS2}$. Bar, 2 µm. **b** knob density and knob diameter. **c** knob heights as determined by electron microscopy. Box plot analyses are overlaid over the individual data points, with the median and the 25% and 75% quartile ranges being shown. Statistical significance was assessed using the Kruskal Wallis one way ANOVA on ranks or the Mann-Whitney rank sum test.

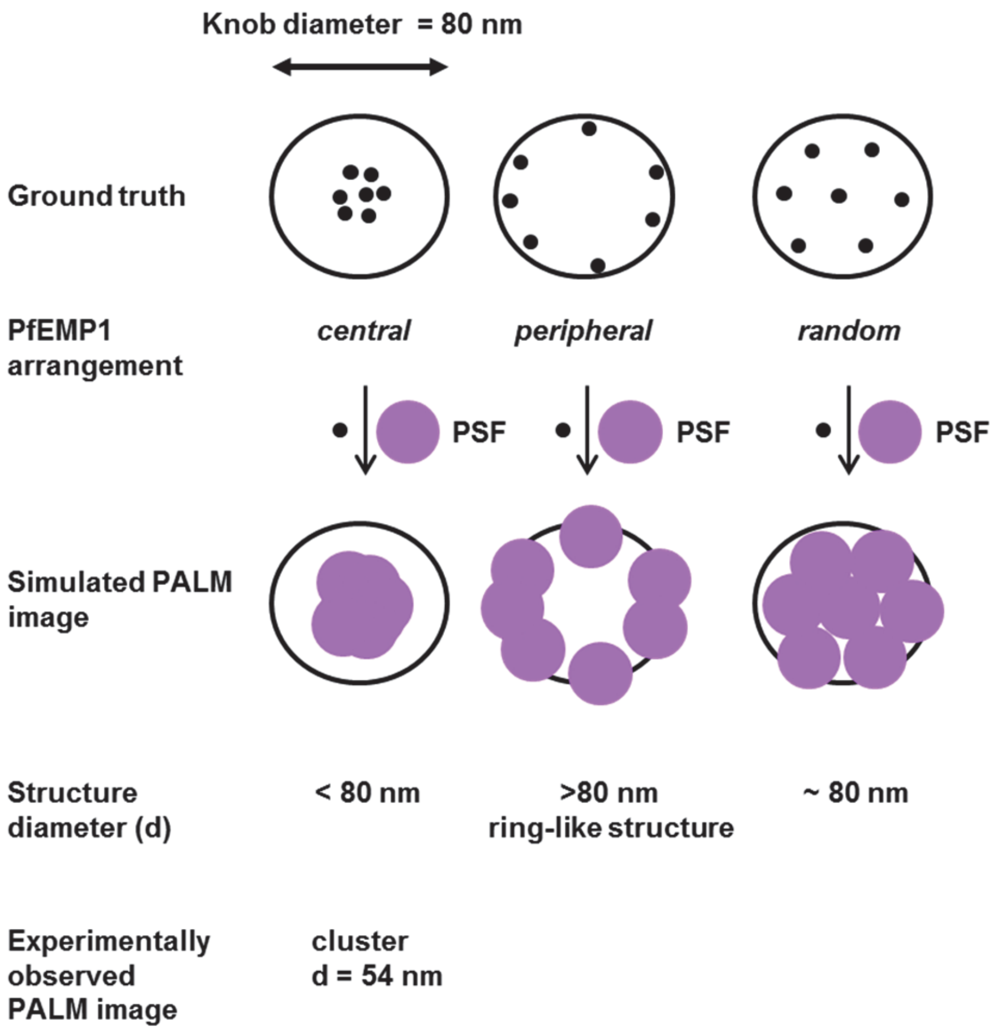

Supplementary Fig. 4 The scheme illustrates the expected imaging results (PALM imaging) for different PfEMP1 clustering architectures within knobs. It takes into account the experimentally derived size of knobs (~ 80 nm, through EM) and the experimental point-spread function (PSF; full width at half maximum FWHM ~20-30 nm) of the PALM microscope. The experimental PALM data obtained for PfEMP1 is in accordance with the first model, in which PfEMP1 associates into clusters that are smaller than knobs.

a

b

c

d

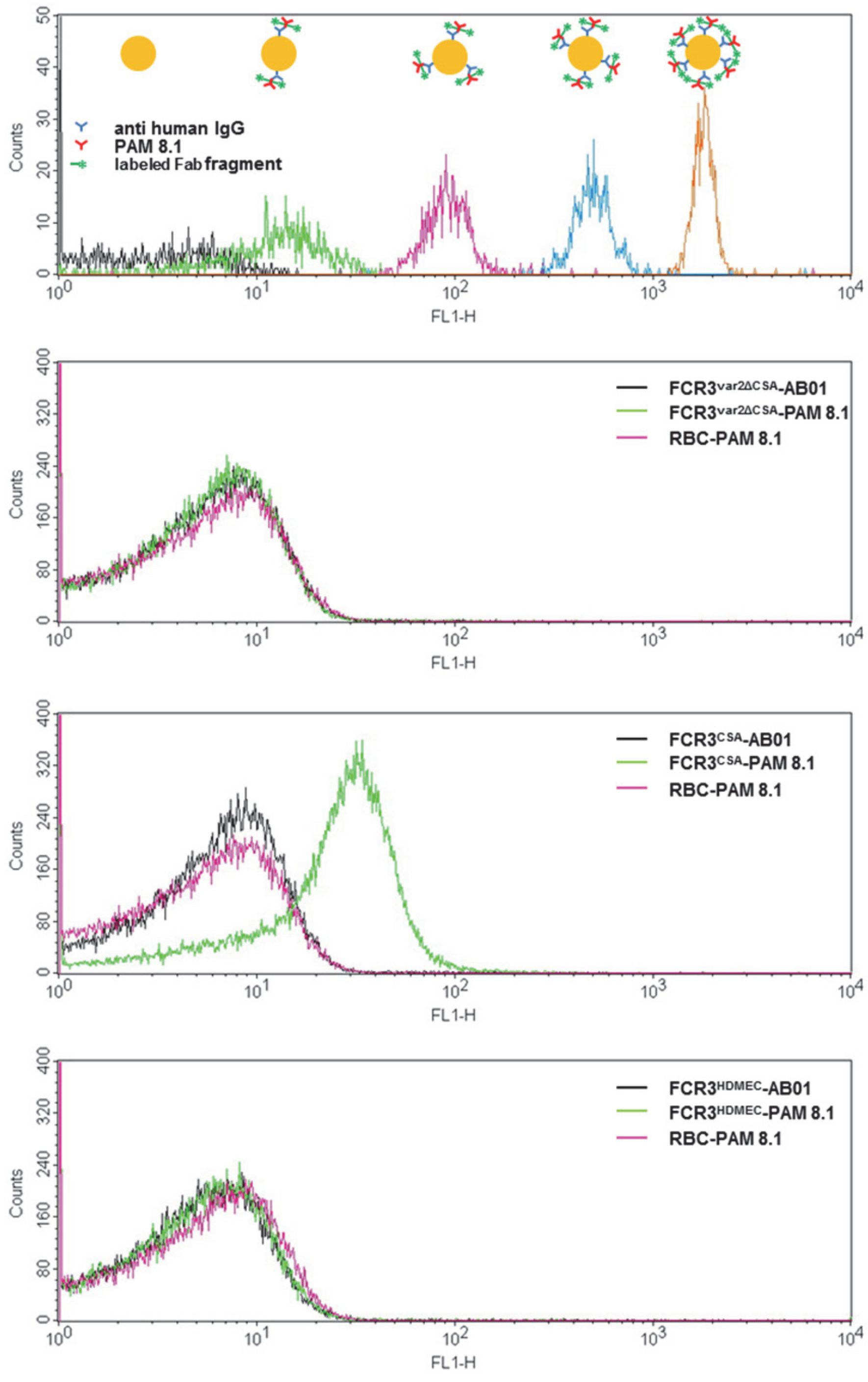

Supplementary Fig. 5 Quantitative FACS analysis for numerical determination of surface-presented PfEMP1 molecules. **a** Calibration of the fluorescence signal. The monoclonal antibodies against VAR2CSA were conjugated with an Alexa Fluor 488 labeled Fab fragment against the Fc domain. These conjugated monoclonal antibodies were subsequently incubated

with microspheres coated with defined amounts of Fc-specific capture antibodies to calibrate fluorescence signals and convert them in absolute molecule numbers. A representative FACS calibration experiment is shown. **b to d** FACS analyses using the cells and antibodies indicated. FCR3^{CSA}, var2csa expressing and CSA binding parasite population; FCR3$^{\Delta var2csa}$, parasite line in which the *var2csa* gene was knocked out by insertional mutagenesis[1]; FCR3^{HDMEC}, FCR3 parasite population panned repeatedly over human dermal microvascular endothelial cells and predominantly expressing IT4var13, IT4var25 and IT4var6, which mediated cytoadhesion to ICAM1 and/or CD36[2]. RBC, uninfected HbAA erythrocytes. Antibodies: the Zeon-conjugated anti-VAR2CSA monoclonal antibody PAM 8.1 and the Zeon-conjugated anti-IT4VAR32b monoclonal antibody AB01[3].

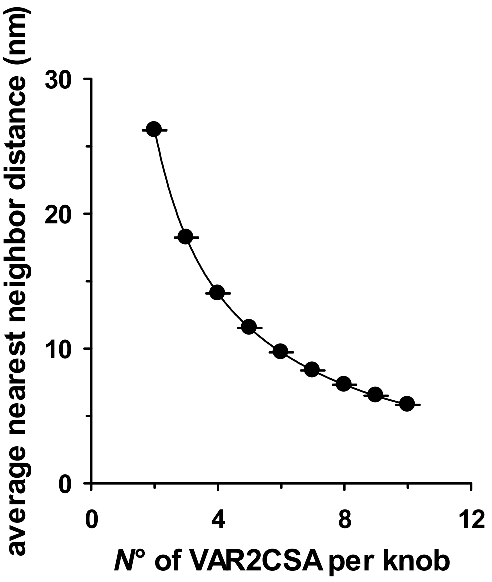

Supplementary Fig. 6 Average nearest neighbor surface-to-surface distance between VAR2CSA molecules on an idealized knob as a function of the number of placed VAR2CSA molecules. An area of 110 nm^2 was considered for a single VAR2CSA molecule[4] such that two molecules do not overlap (see Methods). The model distributed the VAR2CSA molecules according to the calculated density distribution (see Fig. 4d). The mean ± SEM of 20,000 reiterations are shown. A double 5 parameter exponential decay function was fit to the data.

References:

1. Viebig, N. K. *et al.* A single member of the *Plasmodium falciparum var* multigene family determines cytoadhesion to the placental receptor chondroitin sulphate A. *EMBO Rep* **6**, 775-781 (2005).

2. Lansche, C. *et al.* The sickle cell trait affects contact dynamics and endothelial cell activation in *Plasmodium falciparum*-infected erythrocytes. *Communications Biology* **1**, 211 (2018).

3. Barfod, L. *et al.* Human pregnancy-associated malaria-specific B cells target polymorphic, conformational epitopes in VAR2CSA. *Mol Microbiol* **63**, 335-347 (2007).

4. Joergensen, L. M. *et al.* The kinetics of antibody binding to *Plasmodium falciparum* VAR2CSA PfEMP1 antigen and modelling of PfEMP1 antigen packing on the membrane knobs. *Malar J* **9**, 100 (2010).
