# Supplementary material for: Single-molecule imaging and quantification of the immune-variant adhesin VAR2CSA on knobs of Plasmodium falciparum-infected erythrocytes
Source: Commun Biol. 2019 May 8;2:172. doi: 10.1038/s42003-019-0429-z (PMC6506540; doi:10.1038/s42003-019-0429-z)
Supplement: Supplementary file 2 — Reporting Summary [file 42003_2019_429_MOESM2_ESM.pdf]

nature research

Corresponding author(s): Michael Lanzer

Reporting Summary

Nature Research wishes to improve the reproducibility of the work that we publish. This form provides structure for consistency and transparency in reporting. For further information on Nature Research policies, see Authors & Referees and the Editorial Policy Checklist.

Statistical parameters

When statistical analyses are reported, confirm that the following items are present in the relevant location (e.g. figure legend, table legend, main text, or Methods section).

n/a	Confirmed	
☐	☒	The exact sample size (*n*) for each experimental group/condition, given as a discrete number and unit of measurement
☐	☒	An indication of whether measurements were taken from distinct samples or whether the same sample was measured repeatedly
☐	☒	The statistical test(s) used AND whether they are one- or two-sided<br>*Only common tests should be described solely by name; describe more complex techniques in the Methods section.*
☐	☒	A description of all covariates tested
☐	☒	A description of any assumptions or corrections, such as tests of normality and adjustment for multiple comparisons
☐	☒	A full description of the statistics including central tendency (e.g. means) or other basic estimates (e.g. regression coefficient) AND variation (e.g. standard deviation) or associated estimates of uncertainty (e.g. confidence intervals)
☐	☒	For null hypothesis testing, the test statistic (e.g. *F*, *t*, *r*) with confidence intervals, effect sizes, degrees of freedom and *P* value noted<br>*Give P values as exact values whenever suitable.*
☒	☐	For Bayesian analysis, information on the choice of priors and Markov chain Monte Carlo settings
☒	☐	For hierarchical and complex designs, identification of the appropriate level for tests and full reporting of outcomes
☒	☐	Estimates of effect sizes (e.g. Cohen's *d*, Pearson's *r*), indicating how they were calculated
☐	☒	Clearly defined error bars<br>*State explicitly what error bars represent (e.g. SD, SE, CI)*

Our web collection on statistics for biologists may be useful.

Software and code

Policy information about availability of computer code

Data collection	CellQuest Pro; ImageJ; Imaris; Autoquant
Data analysis	Sigma Plot 13; JPK processing software; rapidSTORM; LAMA; DBSCAN

For manuscripts utilizing custom algorithms or software that are central to the research but not yet described in published literature, software must be made available to editors/reviewers upon request. We strongly encourage code deposition in a community repository (e.g. GitHub). See the Nature Research guidelines for submitting code & software for further information.

Data

Policy information about availability of data

All manuscripts must include a data availability statement. This statement should provide the following information, where applicable:

- Accession codes, unique identifiers, or web links for publicly available datasets
- A list of figures that have associated raw data
- A description of any restrictions on data availability

The authors declare that the data supporting the findings of this study are available within the article and its supplementary information files, or are avilable from the authors upon request.

Field-specific reporting

Please select the best fit for your research. If you are not sure, read the appropriate sections before making your selection.

☒ Life sciences ☐ Behavioural & social sciences ☐ Ecological, evolutionary & environmental sciences

For a reference copy of the document with all sections, see nature.com/authors/policies/ReportingSummary-flat.pdf

Life sciences study design

All studies must disclose on these points even when the disclosure is negative.

Sample size	The sample size was calculated using Sigma Plot 13.
Data exclusions	No data were excluded.
Replication	All data reported are based on at least three independent biological replicates. In our case, this means from independent infection experiments using blood from different donors.
Randomization	Samples were not randomly allocated into different groups. This was not possible because we analyzed different developmental stages and different erythrocytes variants.
Blinding	Blinding was not possible. The reason is that we analyzed different developmental stages and different erythrocytes variants.

Reporting for specific materials, systems and methods

Materials & experimental systems

n/a	Involved in the study	
☒	☐	Unique biological materials
☐	☒	Antibodies
☐	☒	Eukaryotic cell lines
☒	☐	Palaeontology
☒	☐	Animals and other organisms
☒	☐	Human research participants

Methods

n/a	Involved in the study	
☒	☐	ChIP-seq
☐	☒	Flow cytometry
☒	☐	MRI-based neuroimaging

Antibodies

Antibodies used	The monoclonal antibodies PAM 8.1, PAM 3.1, PAM 4.7 and AB01 were a gift from Lars Hiviid. The monoclonal antibodies have been described and characterized in Barfod et al. Mol Microbiol 63, 335-347 (2007). The monoclonal antibodies have been validated in the presented study (see Figure 1c and d; and supplementary Figure 5).
Validation	The monoclonal antibodies have been validated in the presented study (see Figure 1c and d; and supplementary Figure 5).

Eukaryotic cell lines

Policy information about cell lines

Cell line source(s)	Human dermal microvascular endothelial cells (HDMEC) were purchased from PromoCell.
Authentication	The human dermal microvascular endothelial cells were authenticated by the manufacturer. In addition, we confirmed surface expression of ICAM-1 and CD36 using specific antibodies to these receptors.
Mycoplasma contamination	All the cell lines were tested negative for mycoplasm contamination, as determined using the Ventor GeM Classic mycoplasm detection kit for conventional PCR from Minerva Biolabs.
Commonly misidentified lines (See ICLAC register)	None were used in this study.

Flow Cytometry

Plots

Confirm that:

☒ The axis labels state the marker and fluorochrome used (e.g. CD4-FITC).

☒ The axis scales are clearly visible. Include numbers along axes only for bottom left plot of group (a 'group' is an analysis of identical markers).

☒ All plots are contour plots with outliers or pseudocolor plots.

☒ A numerical value for number of cells or percentage (with statistics) is provided.

Methodology

Sample preparation	Infected erythrocytes at the trophozoite stage were enriched by magnet purification from a highly synchronized parasite culture. Live cells were labeled using the Zenon-labeled anti-VAR2CSA monoclonal antibody PAM 8.1 (2µg/ml), as described above. Where indicated PAM 4.7 and PAM 3.1, both recognizing the DBL5-ε domain21, were used. The specific fluorescence signals were quantified by FACS analysis using the FACSscalibur (Becton Dickinson). The fluorescence signal was subsequently calibrated and using Quantum™ Simply Cellular (Bangs Laboratories, Inc) as recommended by the supplier and the number of molecules was determinate using QuickCal (Bangs Laboratories, Inc). The FCR3 var2csa knock-out clone and uninfected erythrocytes were analyzed in parallel. The AB01 human monoclonal antibody was used as a negative staining control in parallel assays.
Instrument	FACS Calibur (Becton and Dickinson)
Software	CellQuest Pro
Cell population abundance	All cells were analyzed.
Gating strategy	No gating was performed.

☐ Tick this box to confirm that a figure exemplifying the gating strategy is provided in the Supplementary Information.
